# Genomic variations in patients with myelodysplastic syndrome and karyotypes without numerical or structural changes

**DOI:** 10.1038/s41598-021-81467-2

**Published:** 2021-02-02

**Authors:** Cristiano Luiz Ribeiro, Irene P. Pinto, Samara S. S. Pereira, Lysa B. Minasi, Fernanda de S. M. Kluthcouski, Adriano de M. Arantes, Aparecido D. da Cruz, Marcio A. A. de Almeida, Tom E. Howard, Cláudio C. da Silva

**Affiliations:** 1 Genetics Master’s Program, Replicon Research Group, Department of Agricultural and Biological Sciences, Pontifical Catholic University of Goiás, Rua 235, n. 40, Bloco L, Área IV-S. Universitario, Setor Leste Universitario, Goiânia, GO CEP 74605-050 Brazil; 2 Human Cytogenetics and Molecular Genetics Laboratory, Health Secretary of Goiás State, Goiânia, GO Brazil; 3 Department of Hematology, Clinics Hospital, Federal University of Goiás, Goiânia, GO Brazil; 4 Hospital Araújo Jorge-Cancer Combat Association of Goiás, Goiânia, GO Brazil; 5 Biotechnology and Biodiversity PhD Program, Federal University of Goiás, Goiânia, GO Brazil; 6 Department of Human Genetics, School of Medicine, STDOI-South Texas Diabetes and Obesity Institute, University of Texas Rio Grande Valley, Brownsville, TX USA

**Keywords:** Biotechnology, Cancer, Genetics, Diseases, Medical research, Oncology

## Abstract

Myelodysplastic syndrome (MDS) is an onco-hematologic disease with distinct levels of peripheral blood cytopenias, dysplasias in cell differentiation and various forms of chromosomal and cytogenomic alterations. In this study, the Chromosomal Microarray Analysis (CMA) was performed in patients with primary MDS without numerical and/or structural chromosomal alterations in karyotypes. A total of 17 patients was evaluated by GTG banding and eight patients showed no numerical and/or structural alterations. Then, the CMA was carried out and identified gains and losses CNVs and long continuous stretches of homozygosity (LCSHs). They were mapped on chromosomes 1, 2, 3, 4, 5, 6, 7, 9, 10, 12, 14, 16, 17, 18, 19, 20, 21, X, and Y. Ninety-one genes that have already been implicated in molecular pathways important for cell viability were selected and in-silico expression analyses demonstrated 28 genes differentially expressed in mesenchymal stromal cells of patients. Alterations in these genes may be related to the inactivation of suppressor genes or the activation of oncogenes contributing to the evolution and malignization of MDS. CMA provided additional information in patients without visible changes in the karyotype and our findings could contribute with additional information to improve the prognostic and personalized stratification for patients.

## Introduction

Onco-hematologic diseases constitute a group of neoplasms with different degrees of bone marrow insufficiency, characterized by the expansion of cells with ineffective hematopoietic functions^1,2^. Myelodysplastic syndrome (MDS) comprises a heterogeneous subgroup of neoplasms of bone marrow clones, which present different levels of cytopenia in the peripheral blood, abnormal differentiation of the myeloid lineage, and dysplastic myeloid changes^1,3^. Chromosomal and/or genomic instability inherent in MDS is associated with an increased risk of unfavorable clinical evolution and the development of acute myeloid leukemia (AML), which can occur in 20 to 40% of primary MDS cases^1,4^.

The estimated population incidence rate of MDS is four patients per 100.000 individuals per year in the general population. With advancing age, the incidence of MDS significantly increases. For the age group ≥ 80 years, the incidence of MDS increases 12.5-fold^5^. The average age of onset is 60 years^6^ and only 10% of patients are under 50 years old^5,7^. Chromosomal changes are observed in approximately 50% of primary MDS and 80 to 90% of secondary neoplasms^8–11^^.^ Karyotype data provides important variables for the diagnosis and prognosis of primary MDS, according to the consensus of the International Working Group for the Prognosis of MDS (IWG-PM) of the Myelodysplastic Syndrome Foundation, Inc. The MDS-Foundation proposed the International Prognostic Scoring System Revised (IPSS-R), in which cytogenetic abnormalities are used to predict the overall survival and risk of primary MDS transformation^6^.

The Chromosomal Microarray Analysis (CMA) based on single-nucleotide polymorphism (SNP) is a robust methodology for genomic prospection and, therefore, is useful for investigating cytogenomic rearrangements in MDS^3,12^. The use of CMA could improve MDS diagnosis, refine risk stratification, and provide additional genomic information that can be applied in patient handling and management^13^. The CMA allows for the accurate detection of copy-number variation (CNV) and long continuous stretches of homozygosity (LCSH) acquired in cell-clone neoplasms of MDS patients. This analysis is particularly important in cases where the karyotype was unable to identify chromosomal rearrangements or could not be performed on biological samples^2,14–18^.

Previous studies have implicated LCSHs in the genomic instability observed in patients with MDS^19,20^^.^ LCSHs occur in about 50% of MDS patients with karyotypes without numerical and/or structural alterations. Therefore, genomic variations like LCSH play an important role in tumorigenesis and can alter gene expression^21,22^. CNVs and LCSHs demonstrate prognostic significance in MDS and AML^21^^.^ Previous studies have shown that 74% of the patients evaluated by molecular or cytogenomic techniques present at least one oncogenic copy or pathogenic CNV related to the etiology and development of MDS. Changes in molecular pathways involving epigenetic regulation, chromatin modification, transcriptional regulation, signal transduction, apoptosis, RNA splicing, and cell cycle control in mesenchymal stromal cells in the medullary microenvironment may alter hematopoiesis in MDS^10,23–26^.

In the context previously described, the overall objective of the present study was to identify CNVs and LCSHs by applying the CMA using high-density SNP arrays in patients with primary MDS whose karyotypes presented without numerical and/or structural chromosomal alterations.

## Materials and methods

### Patients and biological materials

This study was conducted at the Replicon Research Center of the School of Agricultural and Biological Sciences of the Pontifical Catholic University of Goiás and in the Human Cytogenetics and Molecular Genetics Laboratory of the Secretary of Goias State for Public Health. The development of this research relied on collaboration with the South Texas Diabetes and Obesity Institute (STDOI), Department of Human Genetics, School of Medicine from the University of Texas Rio Grande Valley in the United States of America. For each of the 17 patients with MDS, the attending physician collected a total of 4 mL of bone marrow tissue. All methods were carried out in accordance with ethical and technical relevant guidelines and regulations, following both national and international consensuses. This study was approved by Human Research Ethics Committee of the Federal University of Goiás Clinics Hospital (HREC-FUG/HC), under protocol no. 1.621.064/2016. All research procedure was done in accordance with the Declaration Helsinki. All patients signed an informed consent document approved by the Human Research Ethics Committee.

### Cytogenetic analysis

Karyotypes from metaphase with a 450-band resolution were obtained through cell culture in bone marrow tissue samples from patients with primary MDS. The chromosomes were banded by the GTG method (following standard procedures), modified, and optimized in accordance with previous protocols^27^. Short-term cultures (48 h) were established by incubating 1.0 mL of cell suspension in 5 mL RPMI1640 (Gibco Life Science, USA) supplemented with 1 mL of fetal bovine serum (Gibco Life Science, USA) and 100 μL L-glutamine (Gibco Life Science, USA) at 37 °C and in 5% CO_2_. To achieve metaphase, mitotic divisions were interrupted by adding 100 μL of colchicine solution (10 μg/mL) in water (Gibco Life Science, USA) to the culture system for 30 min prior to harvesting the cell suspension. Subsequently, the cells were incubated in a hypotonic solution of KCl (0.075 M) (Merck KGaA, Germany) for 30 min at 37 °C and in 5% CO_2_. Following hypotonization, the cells were fixed in methanol solution/glacial acetic acid (3:1), that was added by drip. The cells were mixed, centrifuged, and resuspended in fresh fixative solution. The fixation step was repeated three to four times. The fixed cells were dripped onto microscopy slides, subjected to GTG chromosome banding with the enzyme Trypsin (Invitrogen Life Technologies, USA), and stained in 4% Giemsa solution (Gibco Life Science, USA). Trypsin enzyme was diluted in phosphate-buffered saline (Invitrogen Life Technologies, USA). Twenty metaphases were analyzed per sample. The metaphase images were captured with the aid of an AxioImager 2 microscope (Carl Zeiss, Germany) connected to the blade-sweeping platform Metafer4 (MetaSystems Corporation, Germany). Chromosomal analyses were performed by the software IKAROS (Version 5.0, MetaSystems Corporation, Germany).

### SNP array analysis

The platform GeneChip CytoScan HD array (Thermo Fisher Scientific, USA) was used for the identification of CNVs and LCSHs in the genome of 8/17 patients with primary MDS, who presented karyotypes without numerical or structural alterations. The genomic DNA of bone marrow tissue was extracted using the DNA GenomicPrep Mini Kit (GE Healthcare, USA) following the manufacturer’s instructions. A total of 250 ng of DNA from each sample was digested with the restriction enzyme NspI, following the manufacturer’s recommendations (Thermo Fisher Scientific, USA). Once digested, the samples were connected to the adapters, and then a universal primer was used to amplify the fragments using restriction fragment length polymorphism (RFLP) through polymerase chain reaction (PCR). PCR conditions were optimized to amplify fragments of sizes ranging from 150 to 2000 bp in length. The fragments were confirmed on 2% agarose gel in 1X TBE, subjected to a constant electric field of 10 V/cm for 1 h, and stained in an aqueous solution of ethidium bromide (5 mg/mL). The gel image was captured by the GelDoc XR + Imaging System (Bio-Rad Laboratories, USA).

The amplified products were purified with magnetic beads and quantified on the spectrophotometer NanoVue Plus (GE Healthcare, USA). Next, the amplified products were fragmented into 50- to 200-pb segments, purified, confirmed on a 4% agarose gel in TBE 1X, and separated in a constant electric field of 10 V/cm/h. The bands were revealed in an aqueous solution of ethidium bromide (5 mg/mL). The gel image was captured by the video documentation system GelDoc XR + Imaging System (Bio-Rad Laboratories, USA). DNA fragments were tagged with a terminal deoxynucleotidyl transferase and applied to the GeneChip CytoScan HD, followed by hybridization for 16–18 h at 50 ºC and 60 rpm in the GeneChip Hybridization Oven 645 (Thermo Fisher Scientific, USA). Subsequently, the chips were washed and stained in GeneChip Fluidic Station 450 (Thermo Fisher Scientific, USA) and scanned by a GeneChip Scanner 3000 7G (Thermo Fisher Scientific, USA) controlled by the Thermo Fisher Scientific GeneChip Command Console (AGCC, Version 3.2.2). The array was designed for cytogenomic diagnosis, with approximately a 2.7-million-marker copy number range, covering the entire human genome, including 743,304 SNP markers and > 1.9 million non-polymorphic markers. The CEL archives obtained by scanning each patient were compared with two databases: DGV (Database of Genomic Variants—http://projects.tcag.ca/variation) and CytoScanHD Array Database (provided by software ChAS). CNVs were also compared with the genetic diseases database DECIPHER (Database of Chromosomal Imbalance and Phenotype in Humans using Ensembl Resources: https://decipher.sanger.ac.uk/application/). Additionally, 100% of the constitutional genes were covered by The International Standards for Cytogenomic Arrays Consortium (ISCA: https://www.iscaconsortium.org), including about 12,000 genes from OMIM and more than 36,000 genes from the RefSeq (Reference Sequence Database/NCBI).

In the ChAS, analysis parameters were fixed at 50 markers and a size of 100 kpb to detect gains and at 25 markers and a size of 100 kpb to detect losses. The filter was fixed to ≥ 3 Mb to detect the LCSHs. In the study, the parameters for CNV analysis followed both standard criteria based on manufacture’s recommendations and on the LCSHs analysis previously reported^28,29^. All analyzed chips met the quality control metrics, being MAPD and SNP-QC defined at ≤ 0.25 and ≥ 15, respectively. CNVs, LCSHs, and genes were identified, selected, and separated based on their association with molecular factors of MDS. The databases Online Mendelian Inheritance in Man (OMIM) and RefSeq were accessed by software R to support data mining, analysis, and interpretation of the cytogenomic information obtained by CMA.

### Expression analysis through public databases

Expression analysis was performed on public databases of patients with primary MDS. An NCBI GEO dataset file (https://www.ncbi.nlm.nih.gov/geo/) was downloaded. The study (accession number GSE61853) was based on the analysis of gene expression in mesenchymal tissues of bone marrow stromal cells of patients with MDS and normal controls. The transcriptome was investigated with the aid of the platform Thermo Fisher Scientific Human Gene 1.0 ST Array (USA). Based on the global expression profile of bone marrow mesenchymal stem cells (BM MSCs), the top 250 genes were ranked based on P-value in the study GSE61853 where smallest P-value were the most significant. Afterwards, we compared the genes selected in the CNVs and LCSHs regions with the top 250 genes from the database. The logFC values were used to define which genes were considered to positively or negatively regulated in the individuals with MDS involved in this study.

## Results

**Figure 1:**
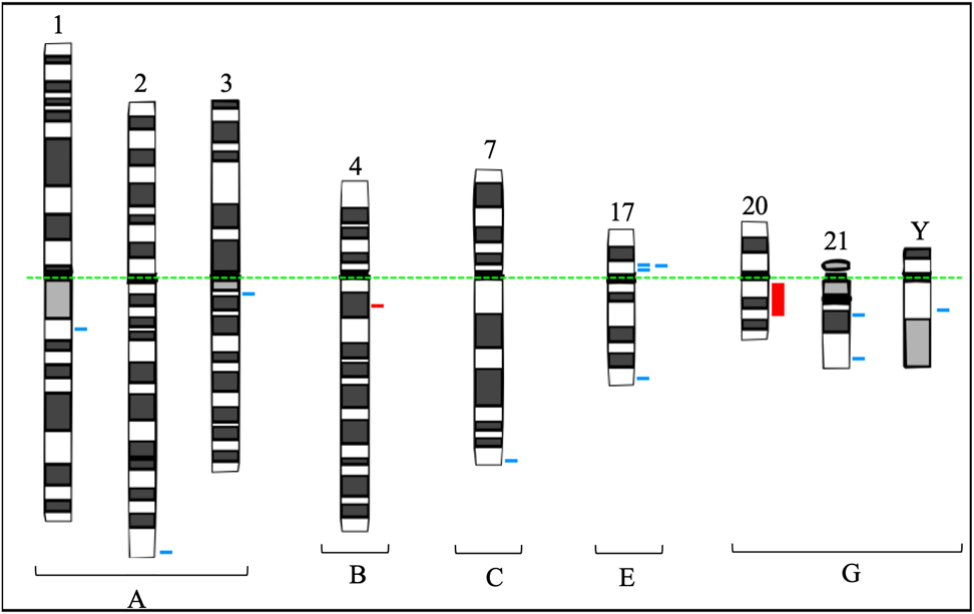
Ideograms of chromosomes following Denver’s group nomenclature summarizing the mapping of 13 unusual and clinically significant CNVs, identified by CMA in 7/8 MDS patients with karyotypes without numerical or structural chromosomal changes. Legend: Blue lines indicate CNVs with genomic gains, while red lines indicate CNVs of genomic loss. A dotted green line was added to indicate the alignment of chromosomes by centromeres.

**Figure 2:**
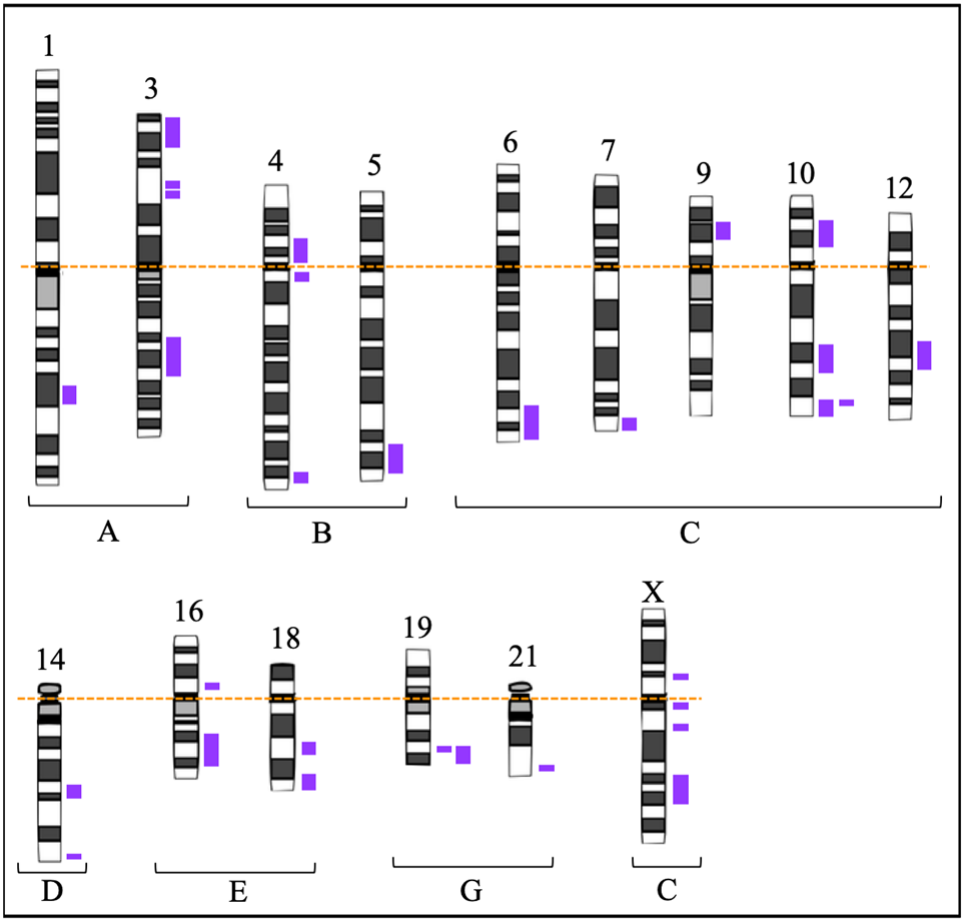
Ideogram of chromosomes following Denver’s group nomenclature summarizing the mapping of 28 unusual and clinically significant LCSHs, indicated by purple bars, identified by CMA in 7/8 patients with MDS and with karyotype without numerical and/or structural chromosomal changes. Legend: A dotted orange line was added to indicate the alignment of chromosomes by centromeres.

The average age of the 17 patients was 56 (26–79) years. The small sample group is a result of the reduced incidence of primary MDS in the population. Of the 17 cases, in 6/17, the results of karyotype with banding GTG demonstrated numerical or structural changes, being + 8, + 15, + 17, + 21, − X, t(4;11)(q21;q23) add(1)(q?), and polyploidy clones; in 3/17, the karyotype was not informative, given that there was no clonal expansion, so these cases were removed from subsequent analyses. In 8/17 patients, the karyotype showed no numerical and/or structural chromosomal changes, and their results were previously reported and were not included in the current analysis^30^. With assistance from CMA, 41 genomic variants were identified in 7/8 patients with no visible changes in karyotype, including 11/41 variations in the number of copies per gain, 2/41 regions with genomic loss, and 28/41 long continuous extensions of homozygosis, mapped on chromosomes 1, 2, 3, 4, 5, 6, 7, 9, 10, 12, 14, 16, 17, 18, 19, 20, 21, X, and Y (Figs. 1 and 2). Ninety-one genes that have already been implicated in relevant molecular pathways for cell viability were selected, including 9/91 gains and 4/91 losses, and 78/91 in regions with LCSHs.

The cytogenetic risk of patients with karyotypes without numerical or structural alterations was rated as good, based on the International Prognostic Scoring System—Revised, while the risk of prognostic transformation of MDS ranged from very low to low. Only one patient died, while the others remained stable at the end of the study. Table 1 summarizes the results identified by the CMA and the risk estimates, according to IPSS-R, in addition to the clinical outcomes of these eight primary MDS patients.

**Table 1:** Clinical features, cytogenetic and cytogenomic results, and risk estimates (according to the International Prognostic Scoring System—Revised) of a cohort of patients with myelodysplastic syndrome in addition to clinical outcomes.

Case	Age (years)	Sex	Cytopenias	Blasts in bone marrow (%)	Karyotypic notation*	IPSS-R** cytogenetic risk	IPSS-R** score	IPSS-R** age-adjusted risk	Risk prediction	Genomic variation	Cytoband	Size (Mb)	Number of genes	Selected OMIN genes***	Clinical outcome
ONCO002	52	F	Thrombocytopenia	2	46,XX^20^	Good	1	0.19	Very low	LCSH	3p26.1	16.9	125	*BHLHE40, LMCD1, RAD18, MTMR14, TADA3, JAGN1, RAF1*	Death
										LCSH	4q11	5.1	40	***	
										LCSH	4p14	10.1	56	*KLHL5, UCHL1, TMEM33, OCIAD2*	
										LCSH	10p13	9.1	47	*CUBN, HACD1, MIR511, MLLT10, BMI1, ARMC3*	
										LCSH	12q21.31	8.7	41	*POC1B, BTG1*	
										LCSH	16p11.2	4.5	65	*RNF40, KAT8*	
										Gain	17p11.2	0.29	4	*MAP2K3*	
ONCO003	49	F	Leukopenia	0	46,XX^20^	Good	1	0.05	Very low	Gain	1q21.3	0.11	5	*CHTOP, ILF2, NPR1, INTS3*	Stable
										Gain	3q12.2	0.1	2	*TFG*	
										Loss	4q13.2	0.12	0	***	
										LCSH	1q31.1	6.88	16	*RGS1*	
										LCSH	4q34.2	10.76	54	*KLKB1*	
										LCSH	6q24.3	23.41	143	*LATS1, ULBP2, ULBP1, DYNLT1, RPS6KA2, AFDN, DLL1*	
										LCSH	10q23.1	15.21	176	*SNORA12, HIF1AN*	
										LCSH	10q26.12	3.9	33	*C10orf88*	
										LCSH	14q22.2	10	70	*BMP4, GMFB, PPM1A, PPP2R5E*	
										LCSH	18q22.3	9.21	38	*ZNF516*	
										LCSH	19q13.32	13.64	602	*ERCC2, SIX5, SYMPK, STRN4, BBC3, BAX, GYS1, CD37, FLT3LG, BCL2L12, IL4I1, SIGLEC7, ZNF446*	
										LCSH	Xp11.23	5.38	108	*GATA1, PIM2, HUWE1*	
										LCSH	Xq13.1	5.65	41	*HDAC8*	
										LCSH	Xq23	14.29	85	*SEPT6*	
ONCO004	42	F	Thrombocytopenia	0.5	46,XX^20^	Good	1	-0.26	Very low	Gain	7q36.3	0.52	3	*PTPRN2*	Stable
ONCO005	57	M	Pancytopenia	0.5	46,XY^20^	Good	2.5	2.01	Low	Loss	20q11.21	17.1	224	*RALY, RAB5IF, ADA, SLC2A10*	Stable
										Gain	21q22.2	0.18	1	*ERG*	
										Gain	Yq11.2	0.41	5	***	
										LCSH	3q22.2	20.2	114	*MRAS, MME*	
										LCSH	9p22.1	9.41	58	*CDKN2B*	
										LCSH	14q32.31	5.45	72	*MOK, TRAF3, TNFAIP2, TRMT61A*	
										LCSH	19q13.32	5.48	244	*ERCC2, SIX5, SYMPK, STRN4, BBC3, BAX, GYS1, CD37, FLT3LG, BCL2L12, IL4I1*	
										LCSH	21q22.3	4.9	120	*MX2, MX1*	
ONCO012	73	F	Thrombocytopenia	0	46,XX^20^	Good	1	1.14	Very low	LCSH	5q33.1	21.1	96	*SPARC, HAVCR2, ITK, TLX3, NPM1*	Stable
										LCSH	7q36.1	4.9	72	*ZNF282*	
										LCSH	10q26.12	10.76	69	*C10orf88, DHX32, DOCK1, MGMT, EBF3*	
ONCO013	42	F	Leukopenia	0	46,XX^20^	Good	1	-0.26	Very low	Gain	2q37.3	0.29	5	***	Stable
										Gain	21q21.1	0.27	4	***	
										LCSH	16q21	17.91	202	*CBFB, NOL3, E2F4*	
										LCSH	Xq11.1	5.19	17	***	
ONCO014	75	F	Thrombocytopenia	0	46,XX^20^	Good	1	1.23	Very low	Gain	17p11.2	0.13	1	***	Stable
										Gain	17p11.2	0.15	2	*SPECC1*	
										Gain	17q25.3	0.15	1	***	
										LCSH	18q21.1	8.19	37	***	
ONCO15	60	F	Pancytopenia	0	46,XX^20^	Good	2.5	2.13	Low	NAF	***	***	***	***	Stable

*F* female, *M* male, *NAF* no alterations found, *LCSH* long continuous stretches of homozygosity.

*ISCN2016: an international system for human cytogenomic chromosome nomenclature.

**IPSS-R: risk estimates, according to the International Prognostic Scoring System—revised

***No cytogenomic rearrangements and no genes were identified using the CMA in the analyzed region.

^#^Estimated score according to hemoglobin levels, absolute neutrophil count, platelet count, percentage of blasts in the bone marrow, and cytogenetic risk.

The size of the genomic changes varied from 0.1 to 23.4 Mb. The LCSHs were larger when compared with the CNVs, ranging from 3.9 to 23.4 Mb. A CNV of loss identified in the cytoband 20q11.21 with 17.1 Mb was the largest genomic variant. The biggest CNV of gain was identified in the cytoband 7q36.3 with 0.52 Mb, while the lowest genomic gain was in cytoband 3q12.2 with 0.1 Mb.

Among the selected genes, 28/91 are differentially expressed by up-regulation or down-regulation in the mesenchymal stromal cells of bone marrow tissue from patients with MDS and karyotypes without numerical or structural changes. The value of logFC was used to define which of the 91 selected genes were regulated positively or negatively in the MDS patients included in this study. Table 2 shows the biological functions and expression patterns of the 28 genes at diagnosis, in addition to the logFC value.

**Table 2:** Biological functions and expression patterns at diagnosis of (28/91) selected genes in MDS patients with karyotypes without numerical or structural changes.

Selected genes	Genomic variation	Cytoband	Biological function*	logFC	Expression**
*MX1* *MX2*	LCSH LCSH	21q22.3 21q22.3	Cellular antiviral response Cellular antiviral response	2.743 1.191	Up-regulation Up-regulation
*POC1B*	LCSH	12q21.31	Centrosome	− 0.522	Down-regulation
*RALY*	Loss	20q11.21	Embryonic development	0.715	Up-regulation
*SIX5* *SYMPK* *ZNF282* *ZNF446*	LCSH LCSH LCSH LCSH	19q13.32 19q13.32 7q36.1 19q13.32	Gene expression control Gene expression control Gene expression control Gene expression control	0.589 1.115 0.392 0.373	Up-regulation Up-regulation Up-regulation Up-regulation
*SLC2A10*	Loss	20q11.21	Glucose homeostasis	− 0.719	Down-regulation
*ADA* *FLT3LG*	Loss LCSH	20q11.21 19q13.32	Immunological response Immunological response	0.696 0.438	Up-regulation Up-regulation
*KLHL5*	LCSH	4p14	Immunological response	0.235	Up-regulation
*UCHL1*	LCSH	4p14	Neuro transmission	− 1.126	Down-regulation
*MAP2K3*	Gain	17p11.2	Oncogene	0.278	Up-regulation
*SNORA12*	LCSH	10q23.1	RNA processing	0.774	Up-regulation
*HACD1*	LCSH	10p13	Signaling molecule	− 0.845	Down-regulation
*TMEM33*	LCSH	4p14	Transmembrane protein	− 0.572	Down-regulation
*TRMT61A*	LCSH	14q32.31	tRNA stabilization	0.593	Up-regulation
*GMFB*	LCSH	14q22.2	Tumor proliferation	− 0.661	Down-regulation
*KAT8* *RGS1*	LCSH LCSH	16p11.2 1q31.1	Tumor proliferation Tumor proliferation	0.681 0.414	Up-regulation Up-regulation
*OCIAD2*	LCSH	4p14	Tumor supressor	− 0.794	Down-regulation
*HIF1AN* *MTMR14* *RNF40* *SIGLEC7*	LCSH LCSH LCSH LCSH	10q23.1 3p26.1 16p11.2 19q13.32	Tumor supressor Tumor supressor Tumor supressor Tumor supressor	0.351 0.367 0.603 0.272	Up-regulation Up-regulation Up-regulation Up-regulation
*C10orf88* *RAB5IF*	LCSH Loss	10q26.12 20q11.21	Identical protein binding Interacting factor protein	− 0.535 − 0.806	Down-regulation Down-regulation

*The biological functions of the genes were identified via RefSeq (NCBI Reference Sequence Database: http://www.genome.jp/dbget-bin/www_bget?ds:H01481; Kegg Disease: Myelodysplastic syndrome—Genome Net).

**For the analysis of expression through public databases of MDS patients, one GEO Dataset from NCBI (https://www.ncbi.nlm.nih.gov/geo/) was downloaded. The transcriptome was based on the study with accession number GSE61853 in which the gene expression patterns of mesenchymal bone marrow stromal cells from patients with MDS and normal controls were analyzed. The value of logFC was used to define which genes were up-regulated or down-regulated in MDS patients with karyotypes without numerical or structural changes.

Some recurrent genomic variations have been identified in patients in the present sample group. The LCSHs in cytoband 19q13.32 with sizes of 13.64 Mb and 5.48 Mb were identified in patients ONCO003 and ONCO005, respectively. In this region, the genes *ERCC2, SIX5, SYMPK, STRN4, BBC3, BAX, GYS1, CD37, FLT3LG, BCL2L12,* and *IL4I1,* which were common among these patients, were selected. That said, the genes *SIX5, SYMPK,* and *FLT3LG* were differentially expressed by up-regulation or down-regulation. The LCSHs in cytoband 10q26.12 with 3.9 Mb and 10.76 Mb were identified in the patients ONCO003 and ONCO012, respectively, and the gene C10orf88 was identified to be differentially expressed by down-regulation. CNVs of genomic gain involving cytoband 17p11.2 with different sizes were also identified. In patient ONCO002 with 0.29 Mb, the up-regulated oncogene *MAP2K3* was selected*,* and in patient ONCO014 with 0.13 Mb and 0.15 Mb, the *SPECC1* gene was selected.

## Discussion

In the present study, patient karyotypes without numerical and/or structural alterations were classified according to cytogenetic risk as good, whereas risk prediction for primary MDS ranged from very low to low. These results confirmed that cytogenetic risk is an important informative variable for inferring prognosis because, according to the clinical outcomes, only one patient died, while the others remained stable at the end of the study. The CMA analysis of patients with primary MDS and no karyotype changes visible under the microscope also provided useful information for better understanding of the etiological aspects of this hematological disease^3,8,10,11^.

CNVs and LCSHs that are potentially associated with the etiology of MDS were identified in 87.5% (7/8) of the patients included in this study, of which 91 genes were selected that had already been implicated in molecular pathways important for cell metabolism and viability. According to the Cancer Genomics Consortium (CGC), CMA shows efficiency in detecting genomic variations in primary MDS in 10 to 80% of patient karyotypes without numerical or structural chromosomal changes^13^. Despite recent advances in diagnostic tools, MDS continues to show variability in its clinical course and response to treatment^1,3,31,32^. In this scenario, the identification of genomic variants associated with primary MDS could prove widely useful, offering additional information for the biological understanding and prognostic classification of this disease^13,15,33,34^.

The imbalances, characterized by gains and losses in the genome, represented 31.7% (13/41) of the genomic variations identified. Therefore, gene copy gains and losses may result in an increase or decrease/absence of any functional transcript. In this context, alterations in gene dosages play a determining role in the quantity of the expressed product and, consequently, down-regulate important molecular pathways of hematopoietic progenitor cells (HPCs), which are associated with the etiological processes of MDS^35,36^.

Expression analysis via a public database indicated that 30.8% (28/91) of the genes in this study located in regions with CNVs and LCSHs are up-regulated or down-regulated in the mesenchymal stromal cells of bone marrow tissue in patients with MDS, when compared with those of controls. Additionally, alterations in gene expression may be related to the inactivation of suppressor genes or the activation of oncogenes^26^. These altered molecular biological mechanisms may interfere with cell survival and correlate with the expansion of cells with ineffective hematopoietic functions, which may contribute to the possibility of the evolution and malignization of clinical MDS.

Of the altered genes, 32.1% (9/28) were down-regulated and 67.9% (19/28) were up-regulated. The main functional pathways in which these genes are involved have been identified; in this context, the investigation only described the biological processes that may be affected by altered gene expression. This study reports that the molecular pathways associated with gene expression control, immune response, and tumor proliferation and suppression were the most commonly deregulated. The relationship between RNA polymerase and the etiology of MDS is not well-defined^26^. One can infer that RNA polymerase dysregulation would alter the normal transcription of critical genes, such as tumor suppressor genes (GSTs), because RNA polymerase plays a role in modulating DNA transcription.

Identified CNVs harbor 17.9% (5/28) of genes with positive or negative regulation in the mesenchymal stromal cells of bone marrow tissue in patients with MDS. In the CNV of genomic loss in 20q, the *RALY* and *ADA* genes were found to be up-regulated and *SLC2A10* and *RAB5IF* were down-regulated. The *ADA* and *RALY* genes are associated with immune response pathways and embryonic development, respectively, while the genes *SLC2A10* and *RAB5IF* correlate with glucose homeostasis pathways and protein interaction factor, respectively; these genes are expressed in the mesenchymal stromal cells of normal bone marrow tissue.

According to reports previously published^37,38^, loss of CNVs lead to haploinsufficiency and can inactivate GSTs. In addition, the *RALY* and *ADA* genes were up-regulated in lost CNVs, suggesting compensation for protein products. Therefore, in microdeletion events in the genome, varied molecular mechanisms can occur and be associated with the pathophysiology of MDS. Thus, CNVs of genomic loss represent important markers with biological significance, providing additional information for understanding the mechanisms and molecular pathways related to the etiology and transformation potential of MDS^36^.

The *MAP2K3* oncogene, located in the CNV with genomic gain in 17p with 0.29 Mb, shows altered expression with positive regulation. The protein encoded by this gene is a dual-specificity kinase that belongs to the family of map-kinases and is activated by mitogenic and environmental stress^38^. Changes in the pathways involving these kinases can deregulate molecular processes related to differentiation, cell cycle survival, and control, leading to the process of apoptosis in patients with MDS. Imbalances caused by positive gene expression, when compared with negative gene expression, may be less unfavorable for cellular biological pathways^37,38^. The present study confirms this statement, since about 2/3 of the genes are up-regulated and 7/8 patients with no visible changes in the karyotype exhibited stable clinical outcomes at the end of the study.

All three gain CNVs involving the 17p11.2 cytoband with different sizes, being 0.29 Mb, 0.13 Mb, and 0.15 Mb, are significant. Events involving chromosome 17p were considered important due to the proximity of the cytoband 17p13.1, which houses the GST *TP53*. The TP53 protein participates in the regulation and expression of several target genes, inducing cell cycle control, apoptosis signaling, cell senescence, and DNA repair^39,40^. Mutations in one or both alleles of *TP53* can deregulate several molecular pathways involved in about 50% of human cancers and 20% of onco-hematologic malignancies, including MDS. Therefore, according to the risk prognosis and clinical results, these CNVs with a 17p gain may have a negative effect on hematological evolution and patient survival, as evidenced by the patient who died during the course of this study. The other CNVs with genomic gain in 2q, 17p, 17q, 21q and Yq, the CNV of genomic loss in 4q, and the LCSHs in 4q, 18q and Xq presented in Table 1 were selected as relevant due to the high instability inherent in the genomes of MDS patients, which is the basis for the accumulation of mutations in this disease^24^. Expression per positive regulation of the *ADA* gene, inserted in a region with genomic loss in 20q, and of the *FLT3LG* gene in 19q and *KLHL5* in 4p, located in regions within LCSHs, may be associated with changes in interferon signaling pathways (immune response)^41^. These deregulated pathways in the medullary microenvironment in patients with MDS demonstrate that these factors participate in inducing the inflammatory state and immunological disorders, which alters hematopoiesis, thereby causing intramedullary apoptosis along with abnormal HPC differentiation and maturation.

LCSHs are abnormalities that represent 68.3% (28/41) of the genomic variations identified in the present study. Of the selected genes, 85.7% (78/91) were inserted in regions with LCSHs and associated with biological regulatory functions. Additionally, 82.1% (23/28) of up-regulated or down-regulated genes in mesenchymal stromal cells in the medullary microenvironment of patients with MDS were located in regions with loss of heterozygosity. The pathological potential of LCSHs emerges as a mechanism of clonal alteration in a proportion of somatic cells with uniparental disomy (UPD)^19,20,42,43^. In this context, recurring areas of LCSHs are strongly associated with the loss of the wild allele or an entire region, which also leads to the hypothesis of haploinsufficiency. Thus, these events indicate changes in biological processes that are important to the cells and signal a predisposition to hematological malignancies^19,20^. The terminal segmental LCSHS identified in 6q, 14q, 18q, 19q, and 21q presented in Fig. 2 indicates that break-induced replication (BIR) can be a dominant mechanism by which a cell duplicates a somatically acquired event, such as a mutation, microdeletion, or epigenetically suppressed region, and consequently becomes homozygous for this segment. BIR seems to be a common repair mechanism in replication, but only the reduplication of a region that contains a genetic or epigenetic alteration gives the cell a growth advantage, allowing for its dominance and clonal selection. Regions with LCSHs involving 3p, 4p, 10p, 12q, and 16p were identified only in the patient who died. The *UCHL1, TMEM33,* and *OCIAD2* genes inserted in 4p, the *HACD1* gene in 10p, and the *POC1B* gene in 12q, which encode neural transmission functions, transmembrane protein, tumor suppressor, signaling molecule, and centrosome, respectively, were down-regulated. Five out of nine selected and down-regulated genes were located in the LCSHs identified in the patient who died. Thus, unlike up-regulation, down-regulation events are potentially more damaging to important molecular pathways and fundamental cellular functions, as observed in this patient during the hematological evolution of the disease^44^.

The *MX1* gene, located in an LCSH region at 21q, was the most up-regulated, being 2.74-times more expressed in bone marrow mesenchymal stromal cells in patients with MDS. This gene encodes a protein that metabolizes the guanosine triphosphate (GTP) that participates in the antiviral cellular response. The protein encoded by this *MX1* gene is induced by the type I and II interferon pathways. In this study, the patients with genomic variations involving 21q were classified as having low and very low predicted risk, respectively, with stable clinical outcomes. These findings contradict previously published^9^ reports that changes involving chromosome 21 have been observed in more advanced cases of MDS, with a more aggressive evolution and rapid leukemic transformation^1,11^. On the other hand, the *UCHL1* gene inserted in an LCSH region with 4p was the most down-regulated, being 1.12-times less expressed in the mesenchymal stromal cells of the medullary tissue of the MDS patient who died. The gene *UCHL1* provides information for the production of the enzyme Ubiquitin Carboxyl-Terminal Esterase L1, which is involved in the cellular machinery that breaks down unnecessary substances^45^. In cells, damaged or excess proteins are marked with ubiquitin molecules so that the ubiquitin–proteasome system can act as a cellular quality control system.

In patients with MDS, persistent and refractory cytopenias can be induced by cytokines and associated with T-cell mediated myelosuppression^46^. Alongside this, changes in the medullary microenvironment inhibit the growth of HPCs through the high secretion of interferon-gamma (IFN-γ), TNF, and Interleukin-6 (IL-6)^14,47^. An increase in the number of stem cells could offset the phenotypic consequences of the simultaneous repression of differentiation genes, creating a pre-leukemic hematopoiesis with little or no morphological abnormalities^48^. As such, the association between dysplastic cell characteristics and the increase in total genomic changes is an observation that suggests a significant parallel trend. Also, the more morphological dysplasia a marrow tissue sample presents, the more extensive the underlying genomic changes can be^48^. Both events, CNVs and LCSHs, contributed significantly to the correlation between the identified genomic variations and the clinical and laboratory phenotypes of patients with MDS in this study, highlighting the usefulness of matrix platforms containing markers for SNPs.

In only 12.5% (1/8) of the patients, the CMA did not reveal genomic variations. It is important to note that this result without changes does not imply that there could not be some kind of deleterious mutation in the genes associated with the molecular pathways associated with the MDS phenotypes. Thus, it was not possible to suggest a genetic cause for this disease, since the CMA resolution was not comprehensive enough to identify changes in the genome. This study highlights that balanced translocations are not detectable by CMA, which is a disadvantage of this cytogenomic analysis technique^49,50^^.^ The possibility of testing other more sensitive or specific genomic technologies, such as Next Generation Exome Sequencing (NGS), may be a differential approach to identify point mutations that may be associated with MDS, in which the etiology is multifactorial and extremely heterogeneous^51^.

The limitation of this study was the small size of the sample group. This limitation is typical of studies involving patients with primary MDS, which have reduced casuistry due to the low incidence of the disease in the population. The present findings from CNVs and LCSHs can contribute to subsequent studies of the adequate characterization of the MDS phenotype and bring additional information to improve the quality of prognostic and personalized stratification for the patient, in addition to the possibility of identifying potentially more effective therapeutic treatments in the near future^13,16^. The interpretation and association of CNVs that result in primary MDS is still challenging, mainly due to the unclear effects of variations in the genome that can be influenced by incomplete penetrance, variable expressiveness, and epistasis^40,52,53^. In this context, further investigations are needed in larger cohorts, including patients with greater heterogeneity of clinical outcomes and prognostic risks.

## Conclusion

This study identified and reported CNVs and LCSHs that may be associated with primary MDS in the group of participants. Ninety-one genes involved in important molecular pathways of metabolism and cell viability were selected. From the analysis of the cytogenomic findings, when compared to information from public genomic databases, CNVs and LCSHs were related to bone marrow failure, which was characterized by the expansion of cells with ineffective hematopoietic functions. CMA is a robust and efficient cytogenomic technique for expanding the resolution of the genomic analysis of the medullary cells of primary MDS patients, allowing the identification of unusual and clinically significant genomic variations in patients without visible changes in the karyotype. Finally, 30.8% (28/91) of the selected genes showed altered expression and 2/3 of these genes showed positive regulation when compared to public data on gene expression in mesenchymal stromal cells of bone marrow tissue in primary MDS. Changes in gene expression may be related to the etiology and progression of primary MDS, with a resulting effect on the relative risk and clinical outcome of this onco-hematological disease.
